# Exposure to mass media and interpersonal counseling has additive effects on exclusive breastfeeding and its psychosocial determinants among Vietnamese mothers

**DOI:** 10.1111/mcn.12330

**Published:** 2016-06-23

**Authors:** Phuong H. Nguyen, Sunny S. Kim, Tuan T. Nguyen, Nemat Hajeebhoy, Lan M. Tran, Silvia Alayon, Marie T. Ruel, Rahul Rawat, Edward A. Frongillo, Purnima Menon

**Affiliations:** ^1^ Poverty, Health and Nutrition Division International Food Policy Research Institute USA; ^2^ Alive & Thrive, FHI 360 Vietnam; ^3^ Save the Children Washington USA; ^4^ Health Promotion, Education, Behavior University of South Carolina Columbia South Carolina USA

**Keywords:** breastfeeding, interpersonal counseling, mass media, psychosocial determinants, Viet Nam

## Abstract

The pathways through which behavior change interventions impact breastfeeding practices have not been well studied. This study aimed to examine: (1) the effects of exposure to mass media and interpersonal counseling on exclusive breastfeeding (EBF) and hypothesized psychosocial determinants (i.e. knowledge, intention, beliefs, social norms, and self‐efficacy); and (2) the pathways through which exposure to mass media and interpersonal counseling are associated with EBF. We used survey data from mothers with children < 2 year (*n* = 2045) from the 2013 process evaluation of Alive & Thrive's program in Viet Nam. Multiple linear regression analyses and structural equation modeling were used to estimate effects. Exposure to mass media only, interpersonal counseling only, both or neither was 51%, 5%, 19% and 25%, respectively. Exposure to both mass media and interpersonal counseling had additive effects on EBF as well as on related psychosocial factors, compared with no exposure. For example, EBF prevalence was 26.1 percentage points (pp) higher in the group that received interpersonal counseling only, 3.9 pp higher in the mass media group and 31.8 pp higher in the group that received both interventions. As hypothesized, more than 90% of the total effect of the two interventions on EBF was explained by the psychosocial factors measured. Our findings suggest that combining different behavior change interventions leads to greater changes in psychosocial factors, which in turn positively affects breastfeeding behaviors.

## Introduction

Exclusive breastfeeding (EBF) up to 6 months of age is recommended to assure optimal health, growth and development of children (Victora *et al*. 2016) and reduce mortality (Sankar *et al*. 2015). Reaching universal optimal breastfeeding could prevent 823 000 annual deaths in children younger than 5 years (Victora *et al*. 2016). Despite these health benefits, the adoption of breastfeeding recommendations remains low in low‐ and middle‐income countries where only 37% of infants are exclusively breastfed (Victora *et al*. 2016). In Viet Nam, the prevalence of EBF is low at 20%, with 16 out of 63 provinces reporting EBF prevalence of 1% or less in 2010, and predominant and partial breastfeeding rates are 26% and 54%, respectively (Nguyen *et al*. 2010; Le *et al*. 2011). Furthermore, EBF prevalence has been stagnant since 2006, alongside increases in early introduction of semi‐solid and solid foods, infant formula use and bottle feeding (Nguyen *et al*. 2011; Gso & Unicef 2014). To reverse these negative trends and promote adoption and continued practice of EBF, effective behavior change interventions at scale are necessary.

Various types of behavior change communication interventions delivered in different settings (e.g. home and family environment, community context, health facilities and mass media) are used to promote optimal breastfeeding practices (Imdad *et al*. 2011; Haroon *et al*. 2013; Sinha *et al*. 2015). Interventions involving face‐to‐face communication, such as interpersonal counseling and group meetings to provide education and/or support for mothers, have been shown to increase EBF (Britton *et al*. 2007; Bhutta *et al*. 2008). Mass media campaigns have also been used to promote individual practices as well as to influence broader social awareness and change, but evidence of their effectiveness is scant (Hernandez *et al*. 1995; Wakefield *et al*. 2010). Findings from a recent review suggest that a combination of interventions (i.e. education and support at the individual and group levels) delivered through different platforms such as the health system and community‐based activities is more effective at improving breastfeeding practices than a single intervention in a single setting (Sinha *et al*. 2015; Rollins *et al*. 2016).

The practice of EBF is influenced by various psychosocial and environmental factors. Modifiable psychosocial factors such as self‐efficacy, confidence, postnatal depression, anxiety, breastfeeding intention, attitudes toward breastfeeding and social support have been shown to be associated with EBF duration, with self‐efficacy being the most predictive (Meedya *et al*. 2010; de Jager *et al*. 2013). Breastfeeding self‐efficacy and comfort with ideas of breastfeeding have also been shown to be positively associated with intentions to breastfeed among expectant mothers, but comfort with formula feeding was the strongest (and negative) predictor of breastfeeding intentions (Nommsen‐Rivers *et al*. 2010). Psychosocial factors have also been shown to be more predictive of EBF duration than socio‐demographic factors (O'Brien *et al*. 2008) such as maternal age, marital status, education level and socio‐economic status (Dennis 2002; McLeod *et al*. 2002), which are less amenable to change. However, no studies to our knowledge have examined the role of psychosocial factors in mediating the impact of programs on the adoption of recommended breastfeeding practices. The present study uses data from Alive & Thrive (A&T) in Viet Nam to assess whether the program interventions improved psychosocial factors hypothesized to be associated with EBF in this context, and whether in turn, the psychosocial factors mediated the program impact on EBF.

A&T is an initiative aimed at improving infant and young child feeding practices (IYCF) in Bangladesh, Ethiopia and Viet Nam through interpersonal counseling and mass media delivered at scale in the context of policy advocacy (Baker *et al*. 2013). In Viet Nam, the program was evaluated using a cluster‐randomized impact evaluation design with repeated cross‐sectional surveys to measure change in the main impact indicators (Menon *et al*. 2013), and a theory‐driven process evaluation to study factors that facilitate or prevent achievement of impact and scale (Rawat *et al*. 2013). We have previously reported the large significant impact of the program on EBF (Rawat *et al*. 2015). Extending beyond the intent‐to‐treat analyses of impact, this paper examines: (1) the exposure of mothers with children 0–24 months of age to the two main interventions – mass media and interpersonal counseling (individually and combined) – and their effects on EBF and hypothesized psychosocial determinants (i.e. knowledge, intention, beliefs, social norms, and self‐efficacy); and (2) the impact pathways through which exposure to mass media and interpersonal counseling are associated with EBF, specifically whether the hypothesized psychosocial factors mediated the effects of the interventions on EBF.

### Program description

In Viet Nam, A&T interventions included three main components: interpersonal counseling on IYCF by trained service providers, mass media campaigns to shape social norms and demand for services and policy advocacy to create an enabling environment for the adoption of optimal IYCF practices. The community‐based component of A&T Viet Nam involved a social franchise model under the brand name *Mat Troi Bé Thơ* (MTBT) (‘The Little Sun’) to provide quality nutrition counseling to women and families at health facilities at all levels. The MTBT management structure, operation and intended service packages have been described in detail elsewhere (Nguyen *et al*. 2013; Nguyen *et al*. 2014). The social franchise model included focused training to build the capacity for providers to give appropriate IYCF advice and support to clients; standardization in service delivery and routine monitoring. (Baker *et al*. 2013). MTBT provided interpersonal counseling and/or group sessions about breastfeeding and complementary feeding practices, beginning in the third trimester of pregnancy and continuing through the first two years of life. Demand for franchise services was generated through the delivery of invitation cards to target groups by village health workers or local nutrition collaborators, a promotional TV spot, distribution of promotional materials and organization of promotional events. MTBT was built on the existing government healthcare infrastructure and its decentralized services to ensure sustainability. Mothers attending the social franchises were provided with knowledge, skills and support including: (1) *knowledge* on the importance of colostrum and early initiation of breastfeeding, the benefit of EBF for the children and mothers, how breastfeeding works, and the risks and hazard of not breastfeeding; (2) skills related to good breastfeeding positioning and attachment; and (3) *support* to identify and address barriers or concerns mothers may have in order to build the mother's confidence in her ability to exclusively breastfeed her baby.

To promote optimal IYCF practices and the use of MTBT services, a mass media campaign was also launched 6 months after initiation of the franchise model. Based on the results from formative research that identified patterns of EBF and its component behaviors, together with data from cultural communication styles, family dynamics, cultural narratives and media engagement, a series of concepts or message platforms were tested. Finally, two television spots on breastfeeding were produced. The first spot, ‘No Water,’ was 30 s long and focused on the importance of not giving water to babies in the first 6 months. The second spot, ‘Nurse More,’ was 45 s long and aimed at building mothers' confidence that they can produce adequate breastmilk with frequent feedings. These two television spots were shown on two national and 15 provincial TV channels and were broadcast in 14 bursts, most of which were 4 to 6 weeks long, from the end of 2011 through November, 2014. The spots also appeared on 24 popular parenting websites in Viet Nam. In addition to the television spots, the mass media campaign included digital and out‐of‐home advertising such as bus wrap advertising, Facebook fan page, mobile phone application and village loudspeaker systems to expose target audiences to key messages.

### Program impact pathway for improving breastfeeding practices

**Figure 1:**
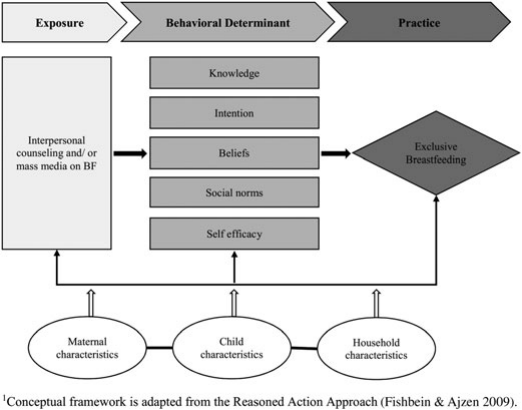
Conceptual framework.

The intended impact pathway of interpersonal counseling and mass media on breastfeeding practices was based on the Theory of Reasoned Action Approach, an integrative framework for the prediction and change of human social behavior (Fishbein & Ajzen 2009). Our adapted conceptual framework specified that exposure to interpersonal counseling and mass media campaign messages leads to a change in maternal knowledge, beliefs about the behavior and potential outcomes of practicing the behavior, perception of social norms and the value placed on these when making decisions, and self‐efficacy about one's own ability to practice these behaviors (Fig. 1). These factors, in turn, influence breastfeeding behaviors. Socio‐demographic factors such as maternal, child and household characteristics may also influence program exposure, psychosocial factors and/or breastfeeding practices.

**Key messages**

- The psychosocial factors (knowledge, intention, beliefs, social norms, and self‐efficacy) played critical roles in mediating the impact of interpersonal counseling and mass media programs on the adoption of recommended breastfeeding practices.
- Combining different behavior change interventions (mass media and interpersonal counseling) leads to greater changes in psychosocial factors, which in turn positively affects breastfeeding behaviors.
- The likelihood of success in future efforts to promote EBF practice will be enhanced through the use of combined interventions to positively influence several psychosocial factors simultaneously with reinforcing messages.

## Methods

### Data sources and study population

This study uses data from the third round of the A&T process evaluation studies in Viet Nam (Rawat *et al*. 2013). The process evaluation studies were conducted within the context of a broader cluster‐randomized impact evaluation for which communes were randomly assigned to be receive either the intensive (including IYCF interpersonal counseling at the franchise facilities, standard government health services and mass media) or non‐intensive (including standard government health services only and mass media) program (Menon *et al*. 2013). For this analysis, data from A&T intensive and non‐intensive areas were merged in order to examine the effects of the two main interventions based on self‐reported exposure (as opposed to intent‐to‐treat). We used the sample of children 0–24 months to examine the association between program exposure and psychosocial factors. For the association with EBF, we used the sample of children 0–6 months because the WHO indicator (EBF under 6 months) is measured using a one‐day recall only among children less than 6 months of age (WHO 2008).

The study took place in four provinces (Thai Nguyen, Thanh Hoa, Quang Ngai and Vinh Long), spanning the northern, central and southern regions of Viet Nam and geographically representative of the 15 provinces where A&T operated. A total of 2045 mother–child pairs participated in the process evaluation survey in 2013 (Rawat *et al*. 2013). Mothers were informed about the purpose of the study, and written informed consent was obtained from all participants. Data were collected via face‐to‐face interviews using a structured questionnaire. Ethical approval was obtained from the Institutional Review Board of the Institute of Social and Medical Studies in Hanoi, Viet Nam and International Food Policy Research Institute in Washington DC, USA.

### Measures

#### Psychosocial and practice variables

The psychosocial variables were classified in five categories: maternal *breastfeeding knowledge*, *intention*, *beliefs* about EBF; *social norms* and *self‐efficacy*. *Breastfeeding knowledge* was assessed based on mothers' answers to a series of questions related to breastfeeding (e.g. how soon after birth breastfeeding should be initiated, benefits of colostrum, benefits of EBF to both mothers and infants, whether mothers should continue breastfeeding if they become sick or pregnant, the ideal duration of breastfeeding, etc.). Some items were previously validated in Haiti (Menon *et al*. 2003) and piloted and adapted for relevance in Viet Nam. Others were added to reflect the program's intervention. Each knowledge item was given a score of 1 (correct) or 0 (incorrect), and the sum was used as the knowledge score (scale: 0 to 27). Other psychosocial indicators were developed by the evaluation team based on the pre‐determined conceptual framework (Fig. 1), proposing that exposure to interventions leads to changes in *intention*, *beliefs*, *social norms and self‐efficacy*. Questions to measure *intention* focused on early initiation and avoidance of any liquids or semi‐solid foods during the first 6 months; question related to *beliefs* included examples of common perceptions of limitations of breastmilk (e.g. child is hungry or thirsty, needs more nutrients) and advantages (benefits for health, nutrition and brain development); questions on *social norms* included statements regarding community/societal support of either EBF or including other liquids/foods before the child reaches 6 months of age and *self‐efficacy* questions included statements regarding the mother's knowledge, capacity and skills to produce enough colostrum, milk, ‘nutrition’ to support her infant's healthy growth and her ability to get support from her family for EBF for 6 months. Each item was measured using a six‐point Likert scale in which women responded the degree to which they agreed or disagreed with several statements (Appendix S1). Responses to negative statements were reverse coded, so all responses were in the same direction as positive items. Cognitive and pilot tests were conducted for all items to ensure that respondents understood the meaning of the survey questions. We constructed total scores for each psychosocial factor by adding the item scores and then standardizing the theoretical range of each scale from 0 to 100 for comparability across factors. The practice variable was *exclusive breastfeeding*, defined as infants 0–5 months of age fed exclusively with breastmilk in the previous 24 h (i.e. no foods or liquids with the exception of medications such as drops and syrups) (WHO 2008).

#### Exposure variables

The main exposure variables were based on self‐reported exposure to mass media and interpersonal counseling. Exposure to mass media considered only exposure to the TV spots, not to other media (i.e. loud speakers, bus wraps, etc.). Mothers were shown four distinct snapshots from the two television spots about breastfeeding and were asked if they had ever seen a television spot with these images. For exposure to interpersonal counseling, mothers were asked if they had ever visited the MTBT franchise service to receive counseling on IYCF practices. Respondents were categorized into four exposure groups: (1) mass media alone; (2) interpersonal counseling alone; (3) both mass media and interpersonal counseling; and (4) neither mass media nor interpersonal counseling.

#### Control variables

Maternal characteristics examined as control variables were age, ethnicity (Kinh majority vs. ethnic minorities), occupation (farmers vs. other jobs) and education (categorized as primary, middle, high school and college or higher). Child‐level variables were child age and gender. We also controlled for household socioeconomic status (SES) which was constructed by principal components analysis of the following variables: ownership of house and land, housing quality, access to services (water, electricity, gas and sanitation services) and household assets (different types of durable goods, productive assets, animals and livestock). The first component derived from component scores (which explained 35% of variance) was then used to categorize the sample into quintiles of SES status (Vyas & Kumaranayake 2006; Gwatkin *et al*. 2007).

### Statistical analysis

Descriptive analysis was used to examine the characteristics of the study sample. Factor analysis was conducted to confirm the structure of the items for *knowledge*, *intention*, *belief*, *social norms and self‐efficacy*. Cronbach alpha coefficients for the five scales ranged from 0.79 to 0.92, indicating adequate internal consistency of items in the scales. These variables were checked for normality using the Kolmogorov–Smirnov test.

Contingency tables and Pearson χ^2^ tests were used to investigate bivariate associations between exposure and EBF. ANOVA tests were used to examine bivariate association between exposure and each of the five psychosocial factors. Multiple linear regression analysis was used to test the association between exposure and EBF (for mothers of children 0–6 months of age), and between exposure and psychosocial factors (for mothers of children 0–24 months of age), after adjusting for maternal (age, ethnicity, occupation and education), child (age and gender) and household characteristics (SES). Structural equation modeling was used to examine the direct and indirect effects of exposure to interventions on EBF through the psychosocial factors. The modeling assumed recursive relationships (i.e. no reverse causality or correlated errors from unmeasured confounders or measurement error). Reverse causality could have occurred if psychosocial factors influenced recall of exposure or if the act of breastfeeding strengthened expression of psychosocial factors, but these reverse paths and uncorrelated errors are likely to have been small given the strong theoretical basis for the modeling. We also checked distributional assumptions, i.e. the normality of univariate variables and the linear bivariate scatterplots between psychosocial factors and exposure. The proportion of mothers reporting EBF was within 0.2 to 0.8, so linear regression was used rather than logistic regression because within this range results are essentially the same (Cox & Snell 1989; Hellevik 2009). Statistical analysis was done using Stata version 13.1 software (StataCorp 2009). Statistical significance was defined as *p* value <0.05.

## Results

Among the 2045 study mothers with children under 2 years old, 87% were Kinh ethnicity, 47% completed at least 9 years of schooling and 41% were farmers. The mean age of mothers was 28 years. The sample of children was 53% boys, and 50% were 0–5 months of age. Scores for breastfeeding knowledge, intention, beliefs, social norm and self‐efficacy were 45, 77, 74, 66 and 72, respectively, out of 100. The prevalence of exposure to mass media only, interpersonal counseling only and both was 51%, 5% and 19%, respectively (Table 1).

**Table 1:** Study sample characteristics

Characteristics	*n*	Percent/mean ± SD
*Dependent variables*		
Maternal knowledge on breastfeeding (range: 0 – 100)	2045	44.74 ± 12.62
Feeding intention (range: 0 – 100)	2045	77.23 ± 18.52
Perceived advantages/disadvantages of breastfeeding (range: 0 – 100)	2045	73.65 ± 16.33
Social norms related to breastfeeding (range: 0 – 100)	2045	65.68 ± 19.24
Self‐efficacy related to breastfeeding (range: 0 – 100)	2045	71.97 ± 18.42
Exclusive breastfeeding among infants <6 mo	1029	50.53
*Independent variables*		
Exposure to Alive &Thrive program components		
None	528	25.82
Reported seeing mass media only	1038	50.76
Reported attending interpersonal counseling session only	94	4.60
Reported both seeing mass media and attending interpersonal counseling session	385	18.83
*Control variables*		
Maternal age, *y*	2045	27.99 ± 5.22
Maternal education, %		
Primary school	160	7.82
Secondary school	923	45.13
High school	505	24.69
College or higher	457	22.35
Maternal occupation as farmer, %	853	41.71
Maternal ethnicity, %		
Ethnic minority	264	12.91
Kinh	1781	87.09
Child gender as male, %	1086	53.11
Child age, %		
0–5.9 mo	1029	49.68
6–24 mo	1016	50.32

Exposure to mass media and interpersonal counseling (individually and combined) was associated with EBF, breastfeeding knowledge, intention, beliefs, social norm and self‐efficacy in the bivariate (Table 2) and multivariate analyses (Table 3). The associations between exposure and outcomes were highest among mothers exposed to both interventions, followed by those exposed to interpersonal counseling alone, and lowest among those exposed to mass media alone. For example, in the model predicting knowledge, the regression coefficients for exposure to both interventions were 14.4, interpersonal counseling only was 11.5 and mass media alone was 2.8. Mothers with higher education and with children < 6 months had higher scores for the psychosocial factors.

**Table 2:** Exclusive breastfeeding prevalence and psychosocial factor scores, by program exposure †All asterisks present the difference from non‐exposure group

	Knowledge	Intention	Beliefs	Social norms	Self‐efficacy	Exclusive breastfeeding
	Mean ± SD	Mean ± SD	Mean ± SD	Mean ± SD	Mean ± SD	Percent
Program exposure						
None	39.91 ± 11.31	69.79 ± 18.94	66.89 ± 16.35	59.19 ± 18.53	65.88 ± 17.90	42.16
Reported seeing mass media only	42.92***, † ± 11.37	76.24*** ± 18.30	72.60*** ± 15.79	64.37*** ± 18.90	70.03*** ± 18.12	45.09
Reported attending interpersonal counseling session only	51.34*** ± 12.12	84.75*** ± 14.72	79.49*** ± 13.30	72.63*** ± 17.24	80.57*** ± 15.02	65.31**
Reported both seeing mass media and attending interpersonal counseling session	54.68*** ± 11.69	88.29*** ± 12.74	84.33*** ± 12.15	76.42*** ± 16.54	83.46*** ± 14.60	73.20***

† All asterisks present the difference from non‐exposure group.

* *P* < *0*.*05.*

** *P* < *0*.*01.*

*** *P* < *0*.*001.*

**Table 3:** Multiple linear regression to examine the association of program exposure with psychosocial factors† and exclusive breastfeeding practice‡

	Knowledge	Intention	Beliefs	Social norms	Self‐efficacy	Exclusive breastfeeding
	β (95% CI)	β (95% CI)	β (95% CI)	β (95% CI)	β (95% CI)	β (95% CI)
Program exposure						
None	Reference	Reference	Reference	Reference	Reference	Reference
Reported seeing mass media only	2.77*** (1.62, 3.92)	6.14*** (4.34, 7.96)	5.26*** (3.68, 6.84)	5.00*** (3.11, 6.91)	4.32*** (2.49, 6.15)	3.93 (−2.83, 10.70)
Reported attending interpersonal counseling session only	11.46*** (9.08, 13.84)	14.98*** (11.23, 18.73)	12.73*** (9.47, 15.99)	13.56*** (9.63, 17.49)	14.62*** (10.84,18.41)	26.08*** (12.14, 40.01)
Both seeing mass media and attending interpersonal counseling	14.39*** (12.96, 15.83)	18.00*** (15.74, 20.26)	16.77*** (14.80, 18.74)	16.53*** (14.17,18.90)	17.37*** (15.09,19.65)	31.79*** (23.38, 40.20)
Maternal characteristic						
Maternal age	0.07 (−0.03, 0.17)	0.14 (−0.01, 0.29)	0.02 (−0.11, 0.16)	0.04 (−0.12, 0.20)	0.12 (−0.05, 0.26)	−0.08 (−0.65, 0.48)
Maternal education						
Primary school	Reference	Reference	Reference	Reference	Reference	Reference
Secondary school	5.12*** (3.24, 6.99)	6.14*** (3.19, 9.09)	6.38*** (3.82, 8.95)	6.72*** (3.63, 9.81)	4.75** (1.78, 7.73)	7.44 (−4.18, 19.07)
High school	7.57*** (5.47, 9.67)	8.05*** (4.74, 11.36)	8.24*** (5.36, 11.12)	9.49*** (6.02, 12.95)	5.86** (2.52, 9.20)	7.84 (−4.99, 20.67)
College or higher	12.05*** (9.83, 14.27)	8.73*** (5.24, 12.23)	9.32*** (6.28, 12.36)	8.63*** (4.97, 12.29)	4.1* (0.57, 7.62)	12.86+ (−0.62, 26.35)
Occupation as farmer	1.25* (0.06, 2.44)	−1.92* (−3.79, −0.04)	−1.07 (−2.70, 0.56)	−0.64 (−2.60, 1.32)	0.29 (−1.60, 2.18)	4.57 (−2.55, 11.69)
Ethnic as Kinh	0.87 (−0.60, 2.34)	1.82 (−0.49, 4.14)	2.56* (0.55, 4.58)	3.48* (1.06, 5.91)	2.27 (−0.07, 4.60)	2.43 (−6.32, 11.18)
Child characteristic						
Child gender as female	0.21 (−0.73, 1.16)	−0.64 (−2.13, 0.85)	−1.06 (−2.35, 0.23)	−1.38 (−2.94, 0.18)	−0.002 (−1.50, 1.50)	0.04 (−5.57, 5.65)
Child age						
0–5.9 mo	3.76*** (2.81, 4.70)	4.85*** (3.36, 6.33)	4.12*** (2.83, 5.42)	6.74*** (5.18, 8.29)	3.81*** (2.31, 5.31)	10.86*** (−12.65,−9.08)
6–24 mo	Reference	Reference	Reference	Reference	Reference	Reference
Household characteristic						
First quintile	Reference	Reference	Reference	Reference	Reference	Reference
Second quintile	0.18 (−1.35, 1.71)	−0.89 (−3.30, 1.52)	−0.23 (−2.32, 1.87)	−0.6 (−3.12, 1.92)	−1.09 (−3.52, 1.34)	−9.41* (−18.44, −0.39)
Third quintile	0.73 (−0.85, 2.30)	1.19 (−1.30, 3.68)	1.08 (−1.09, 3.24)	−0.42 (−3.03, 2.18)	−0.31 (−2.82, 2.20)	−7.47 (−16.85, 1.90)
Fourth quintile	1.42 (−0.27, 3.10)	−0.09 (−2.76, 2.57)	0.82 (−1.50, 3.13)	−0.29 (−3.08, 2.50)	−0.43 (−3.11, 2.25)	−7.98 (−18.09, 2.14)
Fifth quintile	1.51 (−0.29, 3.31)	0.09 (−2.75, 2.93)	0.47 (−2.00, 2.94)	−2.16 (−5.13, 0.81)	−2.27 (−5.13, 0.60)	−11.30* (−22.09, −0.50)

† All psychosocial factors were scored 0 to 100.

‡ Percentage point difference in EBF prevalence between the exposure group and the referent group.

* *P* < *0*.*05.*

** *P* < *0*.*01.*

*** *P* < *0*.*001.*

The total effects of the exposure groups (compared with no exposure) on EBF prevalence were 3.93 (*p* = 0.395) for mass media only, 26.08 (*p* = 0.001) for interpersonal counseling only and 31.79 (*p* < 0.001) for both, indicating that exposure to interpersonal counseling only was associated with a 26 percentage point higher EBF prevalence and exposure to both mass media and interpersonal counseling was associated with a 32 percentage point higher EBF prevalence. In the six separate models predicting EBF and the five psychosocial factors, interactions testing synergy between exposure to mass media and interpersonal counseling were not statistically significant (at *p* > 0.05), suggesting that the exposure effects were additive.

**Figure 2:**
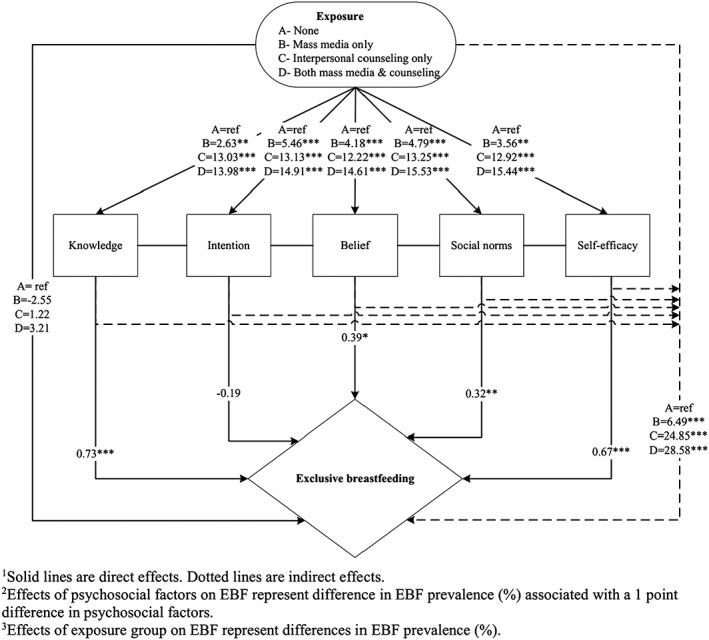
Direct and indirect effects of program exposures on breastfeeding behavioral determinants and exclusive breastfeeding practice.

From the structural equation model, we found that most of the effects of interpersonal counseling only (24.85 pp), and of both mass media and interpersonal counseling on EBF (28.58) was explained by pathways operating through the psychosocial factors (Fig. 2). That is, exposure in these two groups was associated with higher scores for the psychosocial factors, which in turn were associated with a higher EBF prevalence. Pathways through knowledge and self‐efficacy had the largest effects on EBF, followed by social norms and beliefs. Intentions was not significantly associated with EBF, suggesting it was not an important link between exposure and EBF.

## Discussion

Our study results showed that exposure to both mass media and interpersonal counseling was associated with greater knowledge, intention, beliefs, social norms and self‐efficacy about EBF than exposure to either or none of these interventions. Most of these improved psychosocial factors, in turn, were associated with increased EBF practice, a finding that supports the pathways outlined in our conceptual framework. The intention to breastfeed was not observed to be an important link. For each of the outcomes, the association was stronger for exposure to interpersonal counseling only than to mass media only. When both mass media and interpersonal counseling were combined, the associations were additive rather than synergistic. That is, exposure to both interventions produced an effect similar to the sum of the individual effects.

Mass media and interpersonal counseling are distinct interventions that influence psychosocial factors and behavior differently. With the potential to reach large audiences, mass media has been an effective means of communicating preventive health messages at the population level on various health outcomes, including child survival, reproductive health and family planning, HIV/AIDS, smoking and cardiovascular disease prevention (Wakefield *et al*. 2010). Mass media campaigns related to IYCF are more often used to market breastmilk substitutes or other food products than to encourage mothers to exclusively breastfeed (Abrahams 2012; Brady 2012; Piwoz & Huffman 2015). A&T used a mass media campaign not only to promote the benefits of breastfeeding during the first 6 months and highlight the risks of providing water or other breastmilk substitutes, but also to promote positive social norms for EBF. The campaign associated EBF with positive self‐regard, thereby influencing individual knowledge, intention, beliefs, social norms and self‐efficacy related to the practice of EBF.

Exposure to mass media messages is generally passive, however, and may not be sufficiently interactive or context‐specific on its own to create a large change in social norms or rooted cultural habits. In contrast, interpersonal counseling uses face‐to‐face interactions in which health care providers communicate directly with mothers to provide tailored counseling and respond to their expressed needs; interpersonal counseling is intended to affect the decision‐making processes at the individual level. A mother's decision to breastfeed her infant is influenced by counseling and recommendations from healthcare providers (Saadeh 2012), particularly by counselors who are knowledgeable and supportive (Olaolorun & Lawoyin 2006). In addition, community‐based interventions in low‐ and middle‐income countries have been shown to improve EBF at 4 to 5 months (Hall 2011), a critical turning point when mothers often perceive that the child needs to start receiving complementary foods or additional milk in the form of breastmilk substitutes. Consistent with the literature, we found strong associations between interpersonal counseling and EBF as well as the psychosocial factors understood to be determinants of the behavior. Given the strong focus of the social franchise counseling on providing women with knowledge, skills and support for EBF, it is likely that interpersonal counseling in our study was operating through improved maternal capacity which was reflected in greater knowledge and self‐efficacy in our measures.

The lack of association between breastfeeding intentions and EBF in our sample suggests that intention alone may be insufficient to drive behavior change. Multifactorial determinants of breastfeeding require supportive measures at many levels at individual, family, and community health systems and services, and workplace and employment (Rollins *et al*. 2016). Mothers may intend to breastfeed but not be able to practice it in the absence of knowledge and support (both training to support skill development and psychosocial supports like reassurance and social norms) or presence of environmental barriers (e.g. breastmilk‐substitute advertising, poor workplace or employment provisions). In‐hospital formula supplementation has been shown to increase the risk of not fully breastfeeding and early breastfeeding cessation among first‐time mothers intending to EBF (Chantry *et al*. 2014). In our study, the lack of association between intention and the practice of EBF may also be because of the cross‐sectional data and the type of measurement used. The intention to EBF was measured by asking mothers who had an infant between 0 and 6 months of age what their breastfeeding intentions were if they were to have another child. This is different from a more common longitudinal approach to measuring the relationship between intentions and practice, which asks about intentions at one point in time and follows the individuals to measure practices at a second point in time. In our study, mothers who were focused on feeding their young infant may have had difficulties answering questions on their intentions regarding feeding their next child, and this may have been particularly difficult for mothers who at the time did not plan to have another child.

Many individual factors influence breastfeeding practices, including some that may not be modifiable in the short to middle term (e.g. age, marital status, education and income level) and others that are modifiable such as psychosocial factors (e.g. intention, belief, social norms and self‐efficacy) (Meedya *et al*. 2010). The role of psychosocial factors in breastfeeding in general is well‐documented, but there is limited literature on the roles of these factors on EBF in particular (de Jager *et al*. 2013). Intervention studies to date have focused on modifying these factors individually with variable results. A&T was designed to positively influence several psychosocial factors simultaneously, and our results showed that more than 90% of the total effect on EBF practice of interpersonal counseling only and both mass media and interpersonal counseling was mediated by associated changes in the combination of psychosocial factors.

This paper complements our previous paper (Rawat *et al*. 2015) which documents A&T's substantial impacts on increasing EBF practices in Viet Nam. The present paper goes beyond the intent‐to‐treat analyses to further examine the effects of exposure to individual and combined effects of the two main interventions and the pathways linking the interventions to the changes in EBF. Our study data came from surveys of large, cross‐sectional, random samples from both A&T intensive and non‐intensive areas, with good response rates (>95% of estimated sample size). Exposures to mass media and interpersonal counseling were based on self‐reported measures which may be subject to recall bias. In addition, some respondents might have been exposed to the mass media images or logo while attending health facilities for counseling services. If they reported these viewings as exposure to mass media, it could dilute the size of the associations between mass media exposure and psychosocial factors or breastfeeding. Given that mass media exposure is consistently associated with all psychosocial factors, this potential bias does not affect the main conclusions of our study. Measures of breastfeeding practice and psychosocial factors were also from self‐report. These measures may be influenced by recall bias and social desirability bias, if women reported what they thought they should say rather than what actually they did or thought. We addressed this challenge by assessing the role of social desirability in relation to EBF (Reynolds 1982). Although there was some evidence of reporting bias influenced by respondents' desire for social approval, the impact of the A&T program on EBF were still strong even after accounting for social desirability (Menon *et al*. 2015). The accuracy of self‐report related to exposure could also be diminished as the child gets older (e.g. for early initiation and use of colostrum), but we addressed this potential bias by controlling for child age in our models.

Although we acknowledge other potential pathways by which the mass media campaign may have influenced behavior change, we did not explicitly measure these various pathways. For example, mass media messages can initiate and increase the frequency of interpersonal discussion about a particular health issue within an individual's social network, thereby reinforcing changes in behavior. Mass media campaigns can also prompt public discussion of health issues and lead to changes in public policy, creating a favorable environment for individual behavior change. Furthermore, we recognize the bidirectional relationship between the two interventions – the mass media campaign could have prompted visits to the MTBT franchises to receive interpersonal counseling, and the counseling visits also could have led to more attention to and recall of mass media messages.

In conclusion, exposure to mass media and interpersonal counseling was associated with large improvements in psychosocial factors, which in turn were associated with positive breastfeeding behavior. The associations between each outcome and interpersonal counseling only were higher than for mass media only, and the associations were additive, meaning that a mother exposed to both interventions accrued the sum of their benefits. The likelihood of success in future efforts to promote EBF practice will be enhanced through the use of combined interventions with reinforcing messages (Monterrosa *et al*. 2013).

## Source of funding

Funding support from Bill & Melinda Gates Foundation, through Alive & Thrive, managed by FHI360. additional financial support was provided by the CGIAR Research Program on Agriculture for Nutrition and Health (A4NH), led by the International Food Policy Research Institute (IFPRI).

## Conflicts of interest

The authors declare that they have no conflicts of interest.

## Contributions

PHN contributed to the study design, coordinating data collection, developing research questions and conducting the statistical analysis of data, drafting and revising the manuscript; SSK participated in developing the research questions, drafting parts of manuscripts, editing and revising the manuscript; TTN reviewed and provided inputs for data interpretation; NH reviewed and provided inputs for data interpretation; LMT conducting the statistical analysis of data, prepared tables and figures for the manuscript; SA reviewed and provided inputs for data interpretation, and edited the manuscript; MTR contributed to the study design, research questions, and reviewed and edited the manuscript; RR participated in study design and reviewed the manuscript; EAF provided guidance to the statistical analysis and inputs for manuscript, and edited the manuscripts PM contributed to the study design and reviewed the manuscript. All authors read and approved the final submitted manuscript.

## Supporting information


**Appendix S1.** Items used in psychosocial factors for breastfeeding

Supporting info item
