# Supplementary material for: Exposure to mass media and interpersonal counseling has additive effects on exclusive breastfeeding and its psychosocial determinants among Vietnamese mothers
Source: Matern Child Nutr. 2016 Jun 23;12(4):713–25. doi: 10.1111/mcn.12330 (PMC5094561; doi:10.1111/mcn.12330)
Supplement: Supplementary file 1 — Supporting info item [file MCN-12-713-s001.docx]

**Supplementary Appendix**

**Supplementary Appendix 1: Items used in psychosocial factors for breastfeeding**

| **Knowledge^1^** |
| --- |
| Babies should be breastfed immediately or within an hour of birth |
| Babies should be fed colostrum |
| Colostrum protects against allergies and infections |
| Colostrum clear meconium |
| Colostrum helps to prevent jaundice |
| Colostrum helps the intestine to mature |
| Correct definition of EBF (only breastmilk<6 months) |
| Breastfeeding protects baby from illness |
| Breastfeeding helps baby grow and develop better |
| Breastfeeding provides a superior source of nutrients, meets all baby’s nutrient demands in first 6 months |
| Breastmilk easy to digest |
| Breastfeeding provides adequate water for a baby in the first 6 months |
| Breastmilk clean, always ready, and of a good temperature |
| Correct amount of milk a newborn need per feed on the first day after birth (5-7 ml) |
| Correct size of infant’s stomach at 3 days after birth (5-7ml) |
| Mother should empty one breast before switching to the other |
| Breastfeeding whenever baby wants |
| Even women with small breasts can produce enough breastmilk |
| Mother can produce enough breastmilk when she is not fed well |
| Breastmilk alone is better for an infant under 6 months than a combination of breastmilk and infant formula |
| Baby <6 months should not be given water in hot weather |
| Baby <6 months should not be given water to clean the mouth after breastfeeding |
| Mother should continue breastfeeding if she is pregnant |
| Baby <6 months should be fed mother’s expressed breastmilk if mother needs to be away |
| Ideal time to store expressed breastmilk in room temperature |
| Mother should not stop breastfeeding if the mother becomes ill |
| Mothers need to breastfeed the baby until at least 24 months |
| **Feeding intentions^2^** |
| If I have another child, I will not give him/her the following: |
| Any water to drink or to wash out his/her mouth for the first 6 months |
| Anything other than breastmilk in the first 3 days after birth. |
| Any infant formula for the first 6 months |
| Any liquids besides breastmilk for the first 6 months |
| Any solid, semi-solid foods for the first 6 months |
| Any food, water or infant formula for the first 6 months |
| **Beliefs^2^** |
| If I am breastfeeding, but DO NOT give my newborn infant formula during the first 24 hours after birth, s/he will be hungry. |
| If I am Breastfeeding, but DO NOT give my infant water until s/he completes 6 months, my infant will be thirsty. |
| If I feed my infant ONLY breastmilk and NO other food, water or infant formula, until s/he completes 6 months, I am giving my infant all the nutrients s/he needs to be healthy. |
| If I feed my infant a combination of breastmilk and infant formula until s/he completes 6 months, I am giving him/her the BEST possible nutrition. |
| If DO NOT clean my infant’s mouth out with water after breastfeeding, my infant will get thrush. |
| If I am breastfeeding my 5 months old infant, but DO NOT give my infant water, s/he will be too hot. |
| If I am breastfeeding and I wait until my infant has completed 6 mo to start feeding her/him solid, semi-solid foods, it is good for my infant’s health. |
| If I feed my infant a combination of breastmilk and other foods when s/he is between 4 and 6 months of age, I am giving my infant the best possible nutrition. |
| If I feed my infant ONLY breastmilk and NO other food, water, or infant formula until he completes 6 months, I am giving my infant all the nutrients s/he needs for optimal brain development. |
| **Social norms^2^** |
| Most people who are important to me (e.g. family members, friends)… |
| Think that I should feed my infant only breastmilk, and no food, water, or infant formula for the first 6 months. |
| Approve of me giving my baby water before she/he reaches 6 months of age. |
| Approve of me giving my baby infant formula before she/he reaches 6 months of age. |
| Approve of me giving my baby solid, semi-solid foods before s/he reaches 6 months of age. |
| Most women who have infants like me… |
| Feed their infant only breastmilk, and no other food, water, or infant formula for the first 6 months. |
| Give their babies water before they reach 6 months of age. |
| Give their babies infant formula before they reach 6 months of age. |
| Give their babies SSSF before they reach 6 months of age. |
| **Self-efficacy^2^** |
| My body can produce enough colostrum to feed my newborn within one hour after birth. |
| My body can produce enough breastmilk to feed my newborn only breastmilk and no water or infant formula in the first 24 hours. |
| My breastmilk is of good enough quality to nourish my infant so that s/he does not need any other food, water, or infant formula until s/he has completed 6 months. |
| I can refrain from giving my infant water before s/he reaches 6 months of age. |
| I can convince other caretakers of my infant to not give him/her water to drink before s/he reaches 6 months |

^1^Knowledge was assessed based on mothers’ answers (yes/no) to the questions. Each knowledge item was given a score of 1 (correct) or 0 (incorrect).

**^2^** Psychosocial items was measured using six-point scale in which women responded the degree to which they agreed or disagreed with the statement.
